# A Triptych of Care: An Anthropological Interpretation of Morning Bed‐Baths in Greek Intensive Care

**DOI:** 10.1111/nup.70114

**Published:** 2026-08-31

**Authors:** Stelios Parissopoulos, Fiona Timmins, Marianna Mantzorou, George Tsakonitis, Meropi Mpouzika, Eleni Papagaroufali

**Affiliations:** ^1^ Department of Nursing University of West Attica Athens Greece; ^2^ School of Nursing & Midwifery University College Dublin Dublin Ireland; ^3^ Department of Philosophy University of Ioannina Ioannina Greece; ^4^ Department of Nursing Cyprus University of Technology Limassol Cyprus; ^5^ Department of Social Anthropology Panteion University of Social and Political Sciences Athens Greece

**Keywords:** bed‐bath, embodied care, ethnography, intensive care units, nursing care, nursing philosophy, routine care

## Abstract

This paper presents a focused analysis arising from a broader ethnographic study of nursing praxis, power relations, and clinical decision‐making in a Greek ICU. The aim of this paper is to explore the morning bed‐bath as a culturally embedded ICU nursing practice and to interpret how bodily care, professional identity, organisational routines and relational attentiveness intersect within this everyday activity. Informed by critical medical anthropology and phenomenology, the study draws on participant observation, ethnographic fieldnotes, and interviews with intensive care staff. The interpretation draws primarily on Foucault's concept of discipline, complemented by Bourdieu's notion of habitus and embodied practice, and Douglas's work on purity and symbolic order. Three interrelated themes emerged, forming a ‘triptych of care’: a) proximity and synchronicity to the patients' needs and clinical condition, which revealed skilled bodily attentiveness, clinical judgement and nursing agency, alongside negotiations of professional knowledge, and authority; b) the creation of a zone of safety and privacy, where bodily exposure, dignity, relational care, and professional interaction were negotiated within the ICU environment; and c) the removal of dirt and impurities, which served immediate clinical and hygienic purposes while also carrying cultural and professional meanings associated with bodily presentation, cleanliness, and order. The findings suggest that the morning bed‐bath in the ICU studied was not merely a technical nursing task, but a culturally embedded and routinised practice through which temporal and organisational routines, embodied care, symbolic order, and professional nursing values are enacted.

## 1 Introduction

In the high‐technology environment of intensive care units (ICUs), nursing is often defined by continuous clinical vigilance (Kvande et al. 2017), technical proficiency (Alsohime et al. 2021), and the capacity to respond rapidly to critical changes (Holtsmark et al. 2024). However, alongside these acute interventions lies a range of essential routine activities—such as repositioning sedated patients, providing hygiene care, and preparing the bed and body at the start of the clinical day—that remain fundamental to patient safety, comfort, and dignity. These everyday nursing tasks, though vital, are often absent from formal ICU protocols and quality indicators, increasing the risk that they may be inconsistently prioritised or overlooked in practice (Zisberg et al. 2007; Mandal et al. 2020).

Hygiene care in the ICU, and particularly the bed‐bath, plays a critical role in preventing infection, maintaining skin integrity, and supporting physical comfort (Shareef 2021; Coyer et al. 2011; Pentecost et al. 2020; Pols 2013). It also provides a moment of sustained physical contact in a context that is otherwise dominated by technological mediation. Despite this, such care is often framed as ‘basic’ work—less prestigious than technical interventions—and therefore remains underrepresented in both academic literature and policy discussions (Karlsson and Pennbrant 2020). In addition to this, in many healthcare systems, time pressures, staffing shortages, and managerial imperatives have contributed to the routinization of hygiene care, reducing opportunities for individualized, patient‐centred delivery (Griffiths et al. 2019).

A number of studies have shown that ICU nurses’ work is not shaped only by patient acuity or professional judgment (Endacott et al. 2022; Qtait and Sayej 2024; Bergman et al. 2021; Shi et al. 2023). It is also structured by staffing models, workload scoring systems, ICU layout, standardised routines, scheduling and shift design, infection‐control protocols, and broader organizational culture. These institutional priorities and routines create a strong temporal discipline—fast pace, strict deadlines, and high workload—which may influence how nurses prioritise, modify, or ration care.

The aim of this paper is to explore the morning bed‐bath as a culturally embedded ICU nursing practice and to interpret how bodily care, professional identity, organisational routines and relational attentiveness intersect within this everyday activity.

The analysis draws primarily on Foucault (1977) concept of discipline to examine how institutional routines, temporal organisation and repeated practices shape professional conduct within the ICU. However, the interpretation is not exclusively Foucauldian. Pierre Bourdieu's concept of habitus (1977) complements this perspective by drawing attention to the embodied, practical knowledge nurses acquire through repeated participation in clinical practice, while Mary Douglas (1966) work on purity and symbolic order provides a framework for understanding how practices of cleanliness, contamination and bodily presentation acquire cultural and professional meaning within intensive care. Together, these perspectives offer complementary ways of interpreting the morning bed‐bath as simultaneously an institutional, embodied and symbolic practice. While Foucault's framework is useful for illuminating disciplinary structures, its emphasis on institutional power may give less attention to how practitioners exercise agency within, negotiate, or sometimes challenge these structures. These approaches are employed as interpretive lenses rather than comprehensive explanatory frameworks, recognising that each foregrounds particular dimensions of nursing practice while leaving others less visible.

Drawing on Michel Foucault's work on disciplinary power and the ‘medical gaze,’ ICU routines can be understood as mechanisms through which bodies, time, movement, and clinical practices are regulated and made visible within institutional life (Foucault 1973, 1977). In *The Birth of the Clinic*, Foucault described how modern medicine transformed the patient into an object of observation, examination, and intervention under the authority of scientific knowledge, while in *Discipline and Punish* he showed how institutions organise and discipline bodies through routines, surveillance, repetition, and temporal control. One could argue that, within intensive care, these disciplinary processes are reflected in the tightly structured sequencing of clinical activities, the prioritisation of medical timetables, and the regulation of nursing work within the spatial and temporal order of the unit (Melon et al. 2013; Riley and Manias 2002). Although structured temporal schedules can protect against omissions and coordinate clinical work, routines may also reflect institutional priorities and professional relationships that shape when and how nursing care is organised.

For example, across ICU and general acute settings, research consistently shows that when time and staffing are constrained, nurses protect life‐critical tasks and are more likely to delay or omit hygiene, mobilisation, and emotional/psychosocial support. This pattern is well documented in ICU‐specific studies and in broader systematic reviews (Antoszewska and Gutysz‐Wojnicka 2024; Lan et al. 2025; Mandal et al. 2020). This has led to the development of ICU‐specific instruments for assessing missed nursing care, such as the Missed Intensive Nursing Care Scale (MINCS), reflecting increasing recognition of the distinct organisational and relational challenges of critical care environments (Yang et al. 2024).

In some ICUs, the morning bed‐bath is integrated into a highly structured sequence of routine activities, often carried out in successive rounds across the unit. This sequencing serves important organisational functions but can limit opportunities for tailored interaction, patient centred care and responding to patients’ individual needs. Understanding the balance between institutional efficiency and relational care is critical, particularly in the context of changing staffing models and increasing workload pressures in the post‐COVID period (Lake et al. 2022). In the Greek context, ICU nursing operates within resource‐limited settings, with staffing levels and work environments often falling below optimal standards (Moisoglou et al. 2025). Within such conditions, routinised practices may serve important organisational and workload‐management functions, while potentially constraining opportunities for individualised and relational care.

The morning bed‐bath and the way it was performed—known colloquially among nurses in the ethnographic setting as *πρωινό στρώσιμο των αρρώστων* (“*morning bed‐making of the patients*”)—may be considered both a pragmatic response to systemic constraints and a culturally embedded practice that warrants close examination. This is because the bed‐bath unfolds within an organised flow of care activities structured by the temporal rhythms and spatial organisation of the clinical environment (Foucault 1977; Allen 2002; Allen et al. 2002). This structuring of time and space reflects what Foucault (1977) described as the technologies of discipline—systems that regulate bodies, normalize behaviour, and reinforce hierarchies within institutional life. From a Foucauldian perspective, such temporal and spatial organisation may be interpreted as one way through which professional norms and institutional priorities are reproduced. At the same time, the sequencing of morning care may serve practical purposes, including coordinating nursing work, ensuring hygiene care is completed, and preparing patients for subsequent clinical activities such as the medical round.

Ethnographic approaches are well suited to examining everyday nursing routines, as they reveal how institutional structures, cultural norms, and embodied caregiving intersect (Van der Geest and Finkler 2004; Iedema 2004). Focusing on the morning bed‐bath, this study examines how routine hygiene care at an ICU in Greece functions not only as a clinical intervention but also as a site where care, identity, and professional values are negotiated. It contributes to discussions concerning forms of nursing labour that may be less readily represented within dominant biomedical accounts of critical care, highlighting their relational, cultural, and embodied dimensions.

The routinisation of nursing tasks such as the morning bed‐bath can also be considered through Foucault's account of how institutional routines organise clinical time, space, and conduct (Foucault 1977). However, such an interpretation represents only one reading of these practices; routines may simultaneously facilitate coordination, continuity, and the organisation of complex clinical work. The gendered history of bodily and relational nursing work provides an additional context for considering how different forms of nursing activity acquire professional meaning and recognition.

Nevertheless, the strict routinisation of care raises concerns about quality. Though this study took place in the pre‐COVID‐19 period, similar routinisation and ‘task‐team’ care models have since expanded. Such systems may risk emphasising task completion at the expense of some relational dimensions of nursing care (Pattison 2021; Fulbrook et al. 2019). In the context of continuing nursing shortages and changing staffing models, examining how routine care is organised and experienced therefore remains relevant beyond the specific setting of this study.

Ultimately, this paper contributes to debates in nursing philosophy, critical care ethics, and the anthropology of embodiment. It suggests that practices often relegated to the margins of clinical discourse may nevertheless remain central to the everyday enactment of nursing care.

## 2 Design and Methods

### 2.1 Design

This paper presents a focused analysis arising from a broader ethnographic study of nursing praxis, power relations, and clinical decision‐making in the ICU. Within the wider analysis, the morning bed‐bath emerged as a significant and recurrent practice through which multiple dimensions of ICU nursing became visible. The present paper therefore develops an anthropological interpretation of this empirically generated aspect of the wider ethnography.

This study adopts an ethnographic and phenomenologically informed approach to explore nursing practice and nurses’ experiences in a public hospital ICU in Athens, Greece. Drawing on critical medical anthropology and phenomenology, ethnographic inquiry is well suited to examine such routines, revealing how they are enacted, regulated, and made meaningful in everyday practice (Van der Geest and Finkler 2004; Cruz and Higginbottom 2013).

### 2.2 Setting and Informants: Spatial and Temporal Dimension

Fieldwork was conducted over a 2‐year period (2012–2014) in a mixed medical–surgical ICU of a large public hospital. The unit operated up to 18 beds, depending on staffing, and maintained an approximate 1:3 nurse‐to‐patient ratio across all shifts. The patient population included medical, surgical, and trauma cases, with occasional transfers from other specialised units. The ICU also functioned as an educational environment for nursing students, medical residents, and ICU‐trained nurses.

### 2.3 Data Collection: Tools and Methods

Data collection combined participant observation, semi‐structured interviews, ethnographic fieldnotes, and reflexive journaling. Participant observation, aligned with interpretive phenomenology and critical ethnography, focused on the ‘here and now’ of ICU life, capturing embodied practices, social interactions, spatial routines, and interprofessional dynamics within the clinical environment (Hammersley and Atkinson 2007; Emerson et al. 2011; Manias and Street 2001; Sinead Ryan 2017). Ethnographic immersion enabled close engagement with the everyday realities of intensive care nursing and facilitated attention to tacit practices, informal interactions, and culturally embedded dimensions of care that may remain inaccessible through interviews alone.

The first author, an experienced ICU nurse and anthropologist, conducted approximately 250 h of overt participant observation across different shifts in a general ICU in Athens. Field engagement took place regularly over a 2‐year period, with repeated visits to the ICU approximately twice per week across morning, afternoon, and selected night shifts, including both weekdays and weekends. This prolonged and repeated immersion allowed observation of a wide range of routines, interactions, staffing patterns, and organisational rhythms within the unit. Wearing a nursing uniform and participating directly in selected care activities, the researcher adopted the role of participant–observer. Particular attention was paid to bodily care, nurse–patient proximity, touch, temporal routines, clinical urgency, and interprofessional interactions. The morning bed‐bath emerged as a recurring and highly structured practice through which broader organisational, ethical, and relational dimensions of ICU care became visible.

Fieldnotes were expanded into ethnographic ‘scenes’ or vignettes, allowing recurring practices, sensory experiences, and embodied moments of care to be explored in depth. The first author participated in bed‐baths on multiple occasions, assisting nurses in the patient area and recording observations immediately afterwards at a nearby desk. Through sustained immersion in the unit, the researcher was able to observe how bed‐baths unfolded in real time, often shaped by clinical instability, interruptions, staffing pressures, spatial constraints, and interpersonal negotiations among staff.

In addition to participant observation, 20 audio‐recorded semi‐structured interviews were conducted with ICU nurses. Interviews explored participants’ experiences and reflections on caring, touch, routine, visibility, nursing work, and ethical tensions in intensive care. Several participants had been recognised by peers as experienced or ‘expert’ ICU nurses, including nurses working outside the observed ICU setting in educational, managerial, and academic roles. Their inclusion provided additional professional perspectives through which emerging interpretations from the ethnographic setting could be explored and contextualised.

Reflexivity was embedded throughout the research process. The principal investigator maintained a reflective journal and engaged in ongoing critical reflection regarding his dual position as both former ICU nurse and ethnographic researcher. To support analytic rigour and minimise over‐familiarity with the setting, fieldnotes, interpretations, and emerging ethnographic themes were discussed regularly with an academic supervisor from social anthropology. This interdisciplinary dialogue helped challenge assumptions, strengthen critical interpretation, and support broader cultural and social interpretation of ICU practices beyond purely clinical understandings.

### 2.4 Data Analysis

Data were analysed inductively, coded, and grouped into patterns and themes following Braun and Clarke's (2006) six‐stage thematic analysis: a) familiarisation with the data, b) generating initial codes, c) searching for themes, d) reviewing themes, e) defining and naming themes, and f) producing the report. This flexible method, compatible with phenomenological research (Vaismoradi et al. 2013; Nowell et al. 2017), facilitated the categorisation and interpretation of the data. While the broader study included 20 interviews, the findings presented in this paper derive primarily from the ethnographic fieldwork—specifically, sustained participant observation in the ICU—which formed the main source of empirical material. Ethnographic fieldnotes were expanded into detailed ethnographic scenes and organised within the ATLAS.ti v8 software environment. The interviews served a complementary role, providing contextual understanding and triangulation, but the interpretation here is grounded chiefly in the lived, observed practices of the unit.

Data analysis proceeded iteratively and interpretively through repeated engagement with fieldnotes, interviews, reflexive notes, and ethnographic scenes. Initial coding focused inductively on recurring practices, interactions, bodily routines, spatial organisation, and nurses’ narratives of care. As patterns of routinisation, bodily regulation, tacit coordination, symbolic cleanliness, and embodied practical knowledge became increasingly visible across the material, selected social and anthropological theories were used as interpretive lenses to deepen analysis and conceptual understanding. Michel Foucault's work informed the interpretation of institutional discipline, temporal regulation, and routinised bodily practices within ICU care; Pierre Bourdieu's concept of habitus helped illuminate the embodied and practical dimensions of nursing knowledge developed through repeated clinical practice; and Mary Douglas's work on purity and symbolic order informed interpretations of cleanliness, contamination, and bodily presentation in the ICU environment. These theoretical perspectives were not applied deductively as fixed analytical frameworks from the outset; rather, their relevance to the analysis developed progressively through reflexive dialogue between ethnographic observations, participant narratives, and theoretical interpretation.

### 2.5 Rigour—Trustworthiness

Trustworthiness was addressed through credibility, confirmability, dependability, and transferability (Polit and Beck 2017; Nowell et al. 2017; Sandelowski 2004). Credibility was supported by prolonged field engagement, triangulation of data sources, and the inclusion of perspectives from experienced ICU nurses. Transferability was supported by purposive sampling and detailed contextual description of the ethnographic setting and practices, and confirmability/dependability were maintained via a research diary, methodological documentation, and member checks. The iterative nature of ethnographic analysis—moving between theory and data—enabled the identification of tacit knowledge and the development of new insights (Hammersley and Atkinson 2007).

### 2.6 Ethical Considerations

The study received the required institutional ethical approval (Protocol No. 7/11‐7‐12). All participants were fully informed about the aims and nature of the research and provided written consent. Confidentiality was strictly maintained, with all data anonymised. The researcher's dual role as a former ICU nurse and observer was disclosed, and participation in care activities occurred only with prior notification and agreement from staff. ICU staff were informed about the study through posted notices and verbal briefings. Participation was voluntary and overt: nurses who took part in interviews also consented in writing to be observed during their shifts, and only those agreeing to both phases were included in the ethnographic component. Before each observation session, verbal confirmation of willingness to participate was obtained, with reminders that withdrawal was possible at any time. Efforts were made to minimise disruption to daily operations by maintaining an unobtrusive presence. Pseudonyms were used for all participants, and no patient names or identifying details were recorded. Patients were not research participants, and no patient‐identifying information was collected; observations focused on staff interactions and clinical practice.

## 3 Findings

The process of the ICU bed‐bath unfolds through almost identical steps each time it is performed, even if the staff involved changes. This consistency underscores the ritualised nature of the practice: every movement is premeditated, each step has its purpose, and the sequence follows a step‐by‐step pattern known to anyone who has worked in the unit.

[Fieldnote—The morning bed‐bath]

‘It's just after seven o'clock in the morning. The lights come on—they are bright, cold, unrelenting. The patient is stripped of all linen, soiled sheets bundled into laundry bags. Two nurses, side by side, move in unison: they wash him [the patient] head to toe. One nurse on each side. Soaping, rinsing, drying. Again, soaping, rinsing, drying. Their hands are silent, practiced. They do not speak, but the body tells them where to go—chest, arms, armpits, legs, groin. The same ritual unfolds for every patient.’

Bodily care is at the very core of hospital work, and particularly of nursing, to the point that the profession is often defined and distinguished from other health sciences by this intimate engagement with the patient's body. The participant nurses know exactly what to do. This may also be understood as a period in which nurses were the principal professional presence in the patient areas and may reflect nursing preferences and managerial priorities for maintaining standards of patient care.

[Reflective comment]

‘During morning bed‐baths—the so‐called “*bed‐making of the patients*” (adapted from the Greek: το στρώσιμο των αρρώστων)—every nurse's movement is deliberate, every gesture rehearsed through years of repetition. The timing of these steps is known instinctively by those who dwell in this world. While ICU nurse Akrivi [pseudonym] tended to her patient—washing, lifting, soothing—her clinical eye remained vigilant. She assessed the patient as she cleaned, her hands performing a dual role: touch and diagnosis. Her gaze, that expert “nursing gaze,” extended beyond her immediate patient, scanning others across the bay. The ICU patient, once dishevelled *(physically untidy and visibly affected by the night's clinical demands)*, sweaty, and marked by the night's struggles, is transformed. Through this care, they [the patients] become clean, aligned, and visually “ready” for the new day ahead—a body re‐presented to the clinical eye.’

In the ICU observed, bed‐bathing was not marginalised within official unit routines; on the contrary, it was a firmly integrated part of them. However, the unit's protocol‐driven schedule inevitably prioritised medical procedures, meaning that hygiene care, though completed daily and fully, was shaped by the temporal and spatial constraints of other ICU routines. Typically, the aim was to complete the morning bed‐baths by nine o'clock, before the doctors emerged from their morning briefing. If physicians entered the ward during this period, they would remain only briefly to check their patients and prepare for the update meeting. This left nurses alone in the ward for nearly an hour—an uninterrupted stretch of time to carry out the work of bodily care.

[Fieldnotes—Akrivi and Stella washing their patients, Thursday morning]

‘Nurses Akrivi and Stella are washing their patients, change bed linen, and are changing the dressings on pressure ulcers. Along the way, they deal with whatever else arises—calls from doctors, alarms, medications, phone calls. The patient is uncovered; dirty bed linen is placed in a special laundry sack. They work with either a basin of warm water and cloths or pre‐heated moist wipes. They wash from head to toe, one nurse on each side of the bed: soap, rinse, dry… repeat. At times they are very chatty, sharing their observations and news of the day, reassuring and talking to the patient, and sometimes they work in silence; their experienced hands move unerringly—chest, arms, armpits, legs, groin.’

‘The patient is rolled onto their side, revealing reddened skin and a pressure sore. While on the side, they take care not to disconnect the patient from the ventilator or monitor. Saliva runs onto the pillow. Akrivi uses a flexible catheter to suction oral secretions, then another sterile catheter to aspirate bronchial secretions via the endotracheal tube. The patient coughs, triggering the ventilator alarm. They clean the back and buttocks; the patient is not soiled, but has sweated from fever. They change the bottom sheet in two stages—first one half, then the other—turning the patient each time. The body yields, offering no resistance. A pillowcase is changed, secretions suctioned again. The patient is returned to the supine position, the sheet pulled taut over the air mattress, the head and torso elevated.’

‘A final check: arms straight and above the covers, monitor showing normal readings. Satisfied, they cover the patient to just above the chest—no clothing beneath, only the sheet shielding their nakedness. Dirty materials are gathered, gloves and aprons changed, and they move briskly to the next patient, exchanging quick updates with colleagues still bathing others.’

Through this process, the patient—previously sweaty, creased, and marked by the traces of long illness—is transformed into a clean, cared‐for, ‘tidied’ and aligned body, ready for the clinical activities of the day, including the physician's daily examination. The ICU cultural framework normalises this nudity: it is necessary to remove clothes and covers to inspect the skin; it is not misunderstood or sexualised. The linen trolley and the sack for the dirty linen parked in the middle of the unit signal that the ritual is underway and not yet complete.

Looking at nurse Akrivi, her hand and the warm soapy cloth is an extension of her embodied care. Whether gloved or not, her clinical skill, knowledge, and experience are channelled through her fingertips to the patient she treats, whose body is now dependent on others for the most basic functions. As Bradby (2009) notes, nurses work with soiled and vulnerable bodies, routinely breaching the social conventions of Western societies where bodily functions are managed privately and independently. In the ICU, as in other wards, nurses have ‘permission’ to touch, clean, examine, and inspect every part of a patient's skin. In Foucauldian terms, nursing expertise in bodily care, oral hygiene, pressure‐ulcer and wound management may also be understood as a form of knowledge through which nurses acquire particular authority in relation to the care and observation of the patient's body (Foucault 1977).

Nurse Akrivi has worked in the ICU for 7 years, is aged 25–34, and is completing a master's degree in emergency nursing. Regarded as relatively experienced, she is methodical in her approach, skilful in avoiding pain and discomfort, and attentive to detail when managing catheters, cables, and tubes. She is a calm, low‐profile presence with neatly tied hair, minimal make‐up, and clean scrubs, polite with colleagues and patients alike. She avoids open conflicts but will voice—without hesitation—questions or concerns to physicians, even when challenged. As she confided, her main concern was not making errors, but whether she would complete her list of ‘tasks’ before the physicians entered the patient areas—or whether she would be able to cope if something urgent arose. Every morning, bed‐baths began shortly after the morning staff arrived on the unit and received the verbal shift report from the night shift.

[Fieldnote—A Whispered Truth]

‘Akrivi turned to me and said: “We start early because this must be done before they [physicians] come. If not, the head nurse asks ‘why are you behind schedule?’. Some of the doctors don't see what it takes.” Akrivi paused, “No one sees it. But I do.”’

The ethnographic observations suggest that the morning bed‐bath in this ICU was shaped by an interplay of clinical necessity, nursing priorities, staffing constraints, and the temporal organisation of the unit. Although firmly integrated within official nursing routines, bed‐baths were usually completed within a defined morning period before medication rounds, medical rounds, and other clinical interventions increased activity within the unit. This organisation facilitated completion of essential bodily care for all patients, but could also constrain opportunities to individualise its timing and pace. At the same time, the observations revealed that nurses used this period for continuous clinical assessment, embodied observation, and coordination of care.

Beyond its practical function, the organisation of the morning bed‐bath also reflected the temporal and spatial organisation of everyday ICU work. A Foucauldian perspective provides one possible interpretation of how such repeated routines organise time, bodies, and professional conduct (Foucault 1977).

However, the observations also suggest practical and nursing‐led reasons for this organisation. The organisation of bed‐baths into successive rounds had an evident practical function. Completing the baths within the same morning period enabled nurses to provide hygiene care to all patients before the unit became busier with medication rounds, medical rounds, and other clinical interventions. What initially appeared to the researcher as an externally imposed temporal routine could therefore also be understood as a nursing preference and organisational priority arising from the practical demands of caring for multiple critically ill patients.

Oral care was delivered by an auxiliary nurse right after the bed‐baths. The following interaction between the Nurse Manager and nurse Lydia shows how important it was to finish bed‐baths on time and then start oral care.

[Fieldnote—‘the care of the mouths’: Oral care]

Nurse Manager (N.M.): ‘Lydia, are you done with your tasks?’

Lydia: ‘No, I still have the mouths [oral care] to do.’

N.M.: ‘Did you start any of the mouths?’

L.: ‘No, not yet.’

N.M.: ‘But why? What have been doing?’

L.: ‘We finished the bed‐baths at 9:15…’

N.M.: ‘And now? You still have mouths to do? Don't delay… finish this, dear, but don't delay.’

This timing was not explicitly imposed by physicians. Rather, it appeared to form part of the temporal organisation of nursing work within the unit. With a nurse‐to‐patient ratio of approximately 1:3, the early morning provided a practical period in which nurses could complete hygiene care before medication rounds, medical rounds, and other clinical interventions increased demands on their time.

### 3.1 The Triptych of Care

In the ethnographic case, the morning bed‐bath serves as a primary site where ICU nurses develop a more embodied and private relationship with their patients. The triptych of care helps us demonstrate that the bed‐bath in this unit was not only an act of procedural physical care, though the indigenous term ‘bed‐making of patients’ referred to it as such. Through its repeated enactment, the morning bed‐bath not only accomplishes bodily care but also reproduces and makes visible particular understandings of cleanliness, professional competence, organisational order and ‘good’ nursing care.

Ethnographic observations of the morning bed‐bath reveal more than a routine hygiene task: they illuminate the intersection of technical skill, embodied care, and the tacit knowledge that nurses bring to their practice. Within the tightly scripted temporal order of the ICU, such moments reveal multiple dimensions of nursing work—where the body is attended to as both a clinical object and a human presence. The analysis of fieldwork observations forms the first layer of what can be understood as a ***triptych of care*** in the ICU: three interrelated themes that together shape the practice of bed‐baths: a) *Proximity and Synchronicity to Patients' Needs and Clinical Condition*, b) *Creation of a Zone of Safety and Privacy*, and c) *Removal of Dirt and Impurities – Restoration of Bodily Order* (Table 1).

**Table 1:** The triptych of care.

Themes	Description
**1. Proximity and Synchronicity to Patients’ Needs and Clinical Condition**	During the morning bed‐bath, nurses engage in embodied and sensory forms of care, attending closely to patients through touch, movement, observation, and bodily presence. These moments involve heightened attentiveness to patients’ physical condition, comfort, vulnerability, and clinical responses, while also providing opportunities for clinical judgement and nursing agency.
**2. Creation of a Zone of Safety and Privacy**	The bed‐space temporarily becomes a protected relational environment within the wider ICU setting, where nurses provide intimate bodily while seeking to protect patients’ privacy and dignity. Within this space, routine care allows moments of relational attentiveness, professional interaction, and informal learning to emerge.
**3. Removal of Dirt and Impurities—Restoration of Bodily Order**	Morning hygiene care serves immediate clinical and hygienic purposes, while also carrying cultural and professional meanings associated with bodily order, dignity, cleanliness, and presentation. Tidiness and bodily presentation appeared to reflect locally recognised expectations of attentiveness, professionalism, and ‘good care.’

#### 3.1.1 Proximity and Synchronicity to Patients' Needs and Clinical Condition

The bed‐bath brings nurses into physical closeness with the patient's body, requiring intimate handling and the removal of what, within broader cultural and symbolic understandings of bodily contamination, may be perceived as *μιαρό* (“impure” or “unclean”) (Douglas 1966). These moments reveal the skilled, sensory, and affective dimensions of nursing, in which touch, positioning, and bodily management are carried out with practiced precision. At times, depending on the nurse attending to the patient, the bed‐bath unfolded in almost complete silence, with no words needing to be spoken.

[Reflective comment]

‘They [the nurses] don't speak, their experienced hands go where they should and ought to, they know… chest, arms, armpits, legs, groin area.’

‘The silence of the bed‐bath is not emptiness—it is thick with intention. Every swipe of the cloth, every tucked corner, is a statement of presence—not just clinical, but moral, human.’

In this silence, a full sensory relationship unfolds. The hands become instruments of both care and perception. The nurse assesses the patient's ventilation status, secretions, condition of the skin, lines [arterial and central venous], drains and the patient's bodily reactions to movement and stimulation. Is the patient stable? Does the patient cough when turned to change the sheet? Through touch, positioning, and repeated bodily interaction, nurses become highly attentive to subtle clinical and bodily changes. The silence is therefore not an absence, but a mode of concentrated and embodied attentiveness.

On occasions, this embodied attentiveness led experienced nurses to identify and raise concerns about subtle clinical changes that were not immediately apparent through physiological deterioration or other clinical signs. Such observations did not always lead to immediate agreement among members of the interdisciplinary team, but instead initiated negotiations around the interpretation of patients’ conditions and subsequent clinical management. The following ethnographic episode illustrates one such occasion.

[Fieldnote—the displaced endotracheal tube: embodied assessment during the morning bed‐bath]

During the morning bed‐baths, Duchess (pseudonym), one of the unit's most senior ICU nurses, became concerned that the endotracheal tube of a mechanically ventilated patient was not correctly positioned. Based on her own assessment, she believed the tube had already become displaced before she repositioned the patient during the morning bed‐bath and that turning the patient could further compromise its position and ventilation. She immediately communicated her concerns to the medical team and recommended that the tube be replaced. The physicians, however, did not share her assessment at that stage, as the patient remained clinically stable and they could not identify objective clinical signs warranting immediate intervention.

As Duchess [D], one of the nurses, discussed the case with Nektaria (resident physician, N), she questioned whether the tube could have moved while the patient was being turned:

D.: ‘I don't understand… didn't you say you heard the same thing? Are you telling me the tube moved while I was turning the patient?’ Duchess was referring to the audible expiratory air leak that, in her judgement, suggested the tube was no longer correctly positioned.

N.: ‘I said the chest sounds were the same before and after. I didn't say the tube had moved.’

Duchess responded firmly:

D.: ‘Anyway, I've informed you that the tube is like this. I'll document it in the nursing file, and after that you can do whatever you think is best.’

Several minutes later, Duchess called the physician back into the patient's room. Visibly frustrated, she explained to the nurse manager:

D.: ‘When we first examined him this morning, there was an audible air leak and the cuff was completely deflated. We inflated it immediately and I called Nektaria straight away. He hadn't self‐extubated, but this tube is short—it has been cut. As soon as you turn him, it moves. I told them the tube should be changed, but they don't change it. I just don't want them saying that this happened because of us.’

When the nurse manager asked why she felt responsible, Duchess replied:

D.: ‘She said the tube wasn't like this yesterday. I'm telling her it was like this the moment I entered the room. I hadn't even moved the patient. I went straight to get her so she could assess him herself.’

The physicians remained reassuring and continued to monitor the patient, believing that the available clinical findings did not justify immediate tube replacement. The episode revealed a discrepancy between Duchess’ embodied assessment of the patient and the medical team's interpretation of the situation. Rather than abandoning her concerns, she persisted in advocating for the patient while documenting her assessment in the nursing file, thereby fulfilling what she regarded as her professional responsibility.

This episode illustrates that the close bodily proximity established during the morning bed‐bath extended beyond hygienic care to encompass continuous clinical assessment. Through prolonged bodily contact and repeated observation, experienced nurses drew upon embodied clinical knowledge to detect subtle changes in patients’ conditions and advocate for timely intervention. Although their assessments did not always result in immediate agreement within the interdisciplinary team, the vignette demonstrates that nurses exercised professional agency through observation, clinical judgement, documentation and patient advocacy.

One productive way of interpreting these observations is through Michel Foucault (1977) concept of discipline, which helps illuminate how ICU routines become internalised and embodied through repeated practice. Nurses do not require constant verbal instruction during the bed‐bath because the temporal rhythms, bodily movements, priorities, and sequencing of care have already been incorporated into professional conduct through the disciplinary organisation of ICU life. Silence here reflects routinised coordination: nurses communicate through gesture, gaze, touch, timing, and embodied familiarity with both the patient's body and the institutional expectations governing care. Through repeated participation in these routines, nurses develop embodied forms of practical knowledge that enable them to move efficiently within the temporal and spatial order of the ICU while remaining closely attentive to the patient's condition. The preceding vignette further illustrates that this embodied knowledge was not merely reproductive of institutional routines but also informed nurses’ own clinical judgement, enabling them to identify subtle changes, advocate for patients, and actively participate in negotiations surrounding patient care. Within this disciplined structure, bodily care also becomes one of the few moments of sustained proximity and embodied synchronicity between nurse and patient, where attentiveness, ethical presence, and relational care continue to persist despite the highly regulated environment of critical care.

#### 3.1.2 Creation of a Zone of Safety and Privacy

The bed‐bath also creates a temporary protected space within the ward where only those directly involved are present. Within this enclosed micro‐environment, the patient's exposed body is shielded from outside gaze while nurses attend to personal needs. Here, professional exchanges may be interwoven with more personal conversation—telling stories, sharing news, expressing frustrations—over the patient's body. Through these practices, nurses create a temporarily bounded space around the patient, offering a degree of privacy and protection during intimate bodily care within the otherwise open and highly active ICU environment. Under the harsh lights of the unit, this moment becomes one of both vulnerability and protection for the patient.

[Fieldnotes—Beginning the Morning Washes]

‘At 07:15, the unit awakens fully. As I stood at Bed 4, dressed in scrubs, I was no longer merely observing; I was about to assist with the morning washes, something I did each morning I entered the unit for fieldwork. The atmosphere shifted—tense, but not chaotic. The lights revealed everything, while the silence was broken only by the hum of machines. For every six patients, there were two registered nurses and one auxiliary nurse. The night doctor briefly appeared to review the latest arterial blood gases before heading to the meeting room to hand over to the morning medical team. Following a quick exchange of updates—who was stable, who was not—the nurses began the morning bed‐baths. Beside me, a nurse softly greeted her sedated patient: “Good morning.” It was more than habit; it felt almost like an invocation. Although the patient could not respond, the words still mattered. She moved efficiently—uncovering, washing, lifting. Her hands did not flinch at bruises or secretions. Her movements were intimate, practiced, and respectful as she carefully navigated tubes and wires while turning the patient. The bed itself was also attended to—linen smoothed, cables aligned, everything placed in order. She knew, as did I, that this needed to be completed within the organised morning routine, before the unit became busier with medical rounds and other clinical activities.’

[Fieldnote—Learning Without Words]

Around us, others carried out the same ritual. There were no loud instructions, only the quiet, embodied language of care. A junior nurse paused briefly to observe—no words were exchanged, yet learning still occurred. Knowledge here was transmitted through immersion, participation, observation, and presence.

Pierre Bourdieu (1977; 1990) concept of habitus resonates deeply here. Habitus refers to the embodied dispositions, practical understandings, and ways of acting that individuals develop through repeated participation within a social and professional world. In the ICU, nurses perform care through embodied practices developed over years of clinical immersion and repeated participation in routine care—practices not dictated solely by protocols, but by a cultivated practical sense of what the patient needs and how best to respond. Learning occurs not only through formal instruction, but through observation, repetition, bodily proximity, and participation in everyday care practices. Similar ethnographic observations in intensive care have described the rhythms and rituals of ICU work as becoming embedded in professional habits and positionings (Turnbull et al. 2005). This embodied practical knowledge contributes to nurses’ professional identity and enables them to act with confidence and practical competence within the organisational structures of the ICU. Within this temporary zone of care, professional pride and relational attentiveness were also evident, demonstrating how care could remain meaningful, personal, and precise within institutional routines.

#### 3.1.3 Removal of Dirt and Impurities—Restoration of Bodily Order

The restoration of bodily cleanliness was an important component of the morning bed‐bath. Observations suggested that attention to the removal of visible bodily fluids, grooming, clean linen, and the orderly arrangement of the patient and surrounding equipment carried meaning beyond infection prevention and hygiene. Within the unit studied, the presentation of a clean and well‐groomed patient appeared to reflect locally recognised expectations concerning attentive and properly completed nursing care.

[Reflective comment]

‘Grooming and tidying concern the arrangement and control of the patient in bed, the cleanliness of catheters and drains, and the organization of equipment and surrounding space.’

Beyond its immediate hygienic and clinical purpose, the removal of dirt and bodily substances could also be interpreted as contributing to the restoration of bodily and environmental order within the ICU. Mary Douglas's (1966) work on purity and symbolic order suggests that ‘dirt’ is never simply physical matter, but something culturally perceived as out of place and threatening to social order. In the ICU, the removal of bodily fluids, disorder, and visible ‘untidiness’ could be understood not only as practical but as symbolic, restoring a sense of order, control, and professional coherence. From this perspective, nurses’ practices of cleaning, grooming, and tidying could also be interpreted as re‐establishing visual and bodily order within the patient's immediate environment. Such attention to the patient's body and immediate environment also conveyed ongoing care and attentiveness, including towards patients who were sedated or unable to communicate. Within the unit studied, the attention given to cleanliness and bodily presentation also appeared to carry professional meaning: a clean and orderly patient signalled that bodily care had been attended to according to locally recognised expectations of nursing care. Nurses themselves took visible pride in this work.

[Fieldnote—the Head Nurse and Artemis]

On one occasion, after Artemis (A.) had completed a morning bed‐bath with the assistance of Vlassi (V.), an auxiliary nurse, the ICU head nurse (HN) entered the room.

A.: ‘What do you want, boss?’ [emphasis] she called out half‐jokingly.

HN: ‘Can't I check what you're up to myself, guys?’

They laughed, and Artemis teased back:

A.: ‘What do you want? Tell me—*are you checking my patient*? I've made him *koufeto*,’ using the Greek term for the sugar‐coated almonds traditionally handed out at weddings, here meaning ‘beautifully presented’ or ‘perfectly prepared.’

HN: ‘So you washed him yourself? Vlassis [auxiliary nurse] doesn't contribute anything?’

Artemis grinned:

A.: ‘Well, of course Vlassis is a valuable help, no doubt—but **I'm the one** bed‐making [washing] the patient. Could I do this entirely on my own?’

Such interactions highlight not only the pride nurses take in their work, but also how tidiness, bodily presentation, and the organisation of the patient's environment are interpreted by colleagues as indicators of competence, attentiveness, and good care.

The removal of dirt and impurities during the morning bed‐bath therefore had an immediate clinical and hygienic purpose, while the observations also suggest broader cultural and professional meanings associated with cleanliness, bodily presentation, and order. Viewed through Douglas's work, these recurrent practices can be interpreted as contributing to the restoration of bodily and environmental order within this ICU. At the same time, nurses’ attention to the cleanliness and presentation of the patient reflected locally embedded expectations concerning the completion and quality of everyday nursing care.

The cleaning and organisation of the patient's immediate environment therefore served practical and infection‐control purposes, while also expressing care, professional responsibility, and attention to the patient. In this environment of urgency and institutional constraint, nursing presence becomes visible through the consistent and careful enactment of everyday care practices.

## 4 Discussion

This paper presents a focused analysis of the morning bed‐bath, which emerged as a significant practice within the broader ethnographic study of nursing praxis, power relations, and clinical decision‐making in a Greek ICU. The analysis considers the bed‐bath as more than a hygiene procedure, exploring it as a culturally embedded practice through which care, professional identity, organisational routines, and nursing values are enacted in everyday ICU practice.

The three themes identified—proximity and synchronicity to the patients' needs and clinical condition; the creation of a zone of safety and privacy; and the removal of dirt and impurities—highlight the convergence of technical skill, embodied care, and the moral and aesthetic dimensions of nursing work. Within the tightly structured temporal and spatial order of this particular ICU, the morning bed‐bath emerges not only as a clinical necessity, but also as a socially and culturally meaningful practice shaped by institutional routines, symbolic understandings of bodily order, and dimensions of nursing work that may be less readily recognised within formal and technologically oriented representations of ICU care. The findings suggest that these routine acts of bodily care are never merely functional. Rather, they reveal how nurses negotiate attentiveness, dignity, relational presence, and professional pride within highly regulated and technologically intensive environments. The discussion considers how everyday nursing practices reflect and contribute to the professional, relational, and symbolic dimensions of ICU care, drawing selectively on Foucault, Bourdieu, and Douglas as complementary interpretive lenses, alongside scholarship on relational and ethical care in nursing. Through its repeated enactment, the morning bed‐bath not only accomplishes bodily care but also reproduces and makes visible particular understandings of cleanliness, professional competence, organisational order, and ‘good’ nursing care.

The present analysis is informed primarily by a Foucauldian interpretation of institutional routines and disciplinary practices. Alternative theoretical perspectives might have foregrounded other dimensions of the morning bed‐bath, such as collaborative clinical decision‐making, interprofessional coordination, or nurses’ agency. While Bourdieu and Douglas complement the analysis by illuminating embodied practice and symbolic order, the interpretation offered here remains one possible anthropological reading of the ethnographic material.

As for the first theme—**Proximity and Synchronicity to the Patients' Needs and Clinical Condition**—the morning bed‐bath brings nurses into sustained physical closeness with the patient's body, often in a way unmatched by other daily interventions. This proximity is not incidental; it is a form of sustained, intimate labour that involves handling, lifting, repositioning, and the removal of what is considered *μιαρό* (unclean). Nurses’ skilled touch, informed by sensory awareness, ICU‐specific knowledge, and clinical judgement, enables them to attend simultaneously to the patient's hygiene, comfort, and physiological stability. In these moments, the nurse is acutely aware of the patient's vulnerability, *the dependency of the sedated or critically ill body*, and the ethical imperative to preserve dignity. The bed‐bath therefore becomes one of the few moments in ICU care where prolonged bodily proximity and relational attentiveness can occur simultaneously.

Yet, paradoxically, within the organisational frame of the ICU, these acts are often reduced to ‘washing’ or ‘bed‐making of the patients’—terms nurses themselves used to describe these recurrent activities, potentially obscuring the clinical and relational complexity involved in their performance. Foucault (1977) concept of discipline offers one of the ways to understand this paradox. In the ICU, the routinisation of bodily care was embedded within a temporal regime shaped by nursing workload, staffing constraints, medication schedules, medical rounds, and the wider organisational requirements of the unit. The 1:3 nurse‐to‐patient ratio, relatively low for intensive care, limited opportunities for nurses to individualise the timing and pace of care and to deviate from the established temporal organisation of the unit. Bed‐baths generally needed to be completed by approximately 09:00, before activity within the unit increased with medication administration, medical rounds, treatment changes, and new clinical orders. In this context, nurses’ close bodily engagement with patients does not necessarily translate into increased institutional visibility or participation in clinical decision‐making. Studies suggest that the activities nurses perform—or are expected to prioritise—are shaped by broader organisational relations of power, hierarchy, gender, and professional legitimacy (Armstrong 2006; Bourdieu 1977 & 1990; Davies 1995; Foucault 1977; Good 2008).

The bed‐bath is slotted into a tightly organised morning schedule, structured around the wider rhythm of the ICU and the anticipated arrival of physicians for morning rounds. This sequencing reflects what Foucault described as the micro‐physics of power: the organisation of time, space, movement, and bodies in ways that render nursing work efficient, predictable, and aligned with institutional priorities (Mantzoukas and Jasper 2004). At the same time, as the ethnographic observations demonstrated, this sequencing also served practical purposes in coordinating nursing care and managing workload. In this sense, the bed‐bath becomes both a site of relational care and a practice shaped by disciplinary organisation. Nurses may adapt within these constraints—pausing to assess a pressure area, adjusting movements to a patient's tolerance, or responding to subtle bodily changes—but the broader organisational structure could leave limited room for individualised pacing or extended relational engagement.

Nurses worked within established routines that they did not individually determine, but in whose enactment and organisation nursing priorities also played an important role. Each ICU develops its own temporal organisation and rhythm, which new staff members gradually learn to adopt (White 2007; White et al. 2012). The influence of temporal regulation on hospital care has long been recognised in ethnographic studies of clinical work (Strauss and Glaser 1970), where activities such as bathing, repositioning, feeding, and medication administration are organised within tightly structured schedules shaped by institutional demands and time pressures.

This tension between individualised care and institutional routine has also been documented in other ethnographic studies of intensive care (Latimer 2000; McGarry 2007), where bodily care remains central to nurses’ professional identity while simultaneously occupying a marginal position within organisational discourse.

The creation of a temporary zone of privacy and safety during the bed‐bath may also be understood as a space in which nursing agency, embodied knowledge, and dignity‐preserving care were enacted. Within this space, nurses demonstrated attentiveness, embodied presence, and situated clinical knowledge developed through sustained proximity to the patient. At the same time, the ethnographic observations revealed differences in nurses’ participation in formal clinical decision‐making. Several nurses described not knowing the full medical histories of their patients, while during the morning routine nurses and head nurses were occupied with washing, repositioning, assessing, and preparing patients in the ICU rooms as the medical team gathered separately in the seminar room for the formal handover to the day team. These discussions, in which treatment decisions and clinical priorities were reviewed, took place without nurses’ participation. This organisational separation suggests that the situated knowledge nurses developed through continuous bedside care did not necessarily translate into equivalent participation in formal medical decision‐making. Nevertheless, as other observations in this study demonstrate, nurses were not passive within these professional relationships: they exercised clinical judgement, communicated their assessments, negotiated with medical colleagues, and advocated for patients within the interdisciplinary team.

Turning to the second theme—**Creation of a Zone of Safety and Privacy**—we see a different dimension of the bed‐bath's significance. The act creates a temporary spatial and social boundary within the otherwise open, surveyed environment of the ICU. Curtains are drawn, doors partially closed, and the small team performing the bath occupies an enclosed micro‐environment where only those directly involved enter. Within this space, the patient's body is simultaneously exposed—to those providing care—and protected—from the gaze of outsiders. The harsh lighting of the ICU falls on the patient's skin, but the surrounding world is, for a time, kept at bay. In a setting characterised by constant observation, alarms, interruptions, and clinical urgency, the bed‐bath briefly transforms the bedside into a more contained and relational space of care.

Bourdieu (1977) notion of habitus and embodied practice helps to explain the social dynamics of this zone. The nurses’ movements, their sequencing of tasks, and even their conversational patterns are products of a deeply ingrained professional habitus—acquired through training, repetition, and adaptation to the ICU's culture. Within the enclosed space of the bed‐bath, this habitus governs not only how the body is handled but also how nurses interact with one another. They exchange clinical observations, share personal stories, and, at times, voice frustrations—often over the patient's inert body. These moments reveal the dual nature of the bed‐bath space: it is simultaneously a site of intimate bodily care, informal learning, peer interaction, and tacit knowledge exchange.

Lake et al. (2022), in applying Bourdieu's theory to nursing, argue that care practices emerge from socialised dispositions that are both individual and collective. Nurses, particularly in highly regulated environments such as the ICU, develop shared embodied understandings that guide their actions within institutional constraints. These forms of practical knowledge are learned not only through formal education, but also through observation, repetition, bodily proximity, and participation in everyday routines. Viewed through Bourdieu's understanding of practice, the routinised character of ICU work does not necessarily imply passive conformity. Nurses remain active agents whose embodied practical knowledge enables them to interpret clinical situations, exercise judgement, and negotiate their actions within the organisational and professional structures in which practice occurs. The private zone of care described in our fieldnotes is one of the spaces where this embodied knowledge becomes most visible. Within the broader organisational structure of the ICU, the bedside becomes a micro‐environment where nurses enact professional identity through routine bodily practices. The careful positioning of sheets, the management of catheters and lines, and the organisation of the patient's immediate environment reflect not only technical competence but also attentiveness, relational presence, and professional pride.

At the same time, this embodied knowledge frequently operates within established professional and organisational relationships in the ICU. While ICU nurses are expected to monitor patients continuously and contribute to medical decision‐making, previous studies have suggested that nurses may have limited involvement in formal decision‐making processes (Papathanassoglou et al. 2012).

Our observations add a more nuanced perspective. Nurses demonstrated considerable embodied expertise and clinical judgement during bedside care and, as illustrated in the endotracheal‐tube vignette, could actively communicate, negotiate, and advocate for their assessments within the interdisciplinary team. The findings therefore suggest that nursing knowledge was not simply invisible or powerless, but that its recognition and influence were negotiated within the professional and organisational relationships of the ICU. Routine bodily care activities such as bathing, oral hygiene, repositioning, and maintaining patient comfort are essential components of intensive care nursing, yet their complexity and contribution may be less readily recognised within formal representations of ICU work, where technologically oriented and biomedical interventions are often more readily visible.

Nevertheless, the creation of this private zone also has an important moral dimension. By shielding the patient from unnecessary exposure, nurses enact forms of protection and respect that align with ethical understandings of dignity in care (Gallagher 2024; Gastmans et al. 1998). Yet this privacy is negotiated within a context where patients may be sedated or unconscious, unable to verbally express preferences or discomfort. Nurses therefore draw on embodied and tacit forms of moral reasoning when deciding how much of the body to uncover, how to position sheets, and when to pause if another staff member enters the room. These actions are rarely formalised or explicitly discussed, yet they constitute an important ethical dimension of everyday nursing practice.

The findings of the present study align with Vouzavali et al. (2011) phenomenologically informed ethnography, which emphasised the embodied and relational dimensions of nursing care in intensive care settings. Their interpretation of the nurse–patient encounter as spatial, moral, and deeply corporeal resonates with the ‘zones of privacy’ described in our ICU observations, where nurses created brief but meaningful moments of individualised care despite the rigid and technologically saturated environment. In both studies, touch, presence, and spatial proximity function not only as clinical necessities but also as ways of preserving dignity and relational attentiveness. Our findings extend this interpretation by showing how these moments emerge within highly routinised morning care practices, where nurses negotiated institutional demands while simultaneously attempting to preserve the patient's bodily integrity and personhood.

Our study also supports research conducted in other high‐intensity clinical settings, where privacy is often fragile, temporary, and actively negotiated (Lawler 1991; Turnbull et al. 2005). In our ICU, the morning bed‐bath represented one of the few predictable opportunities for nurses to create and maintain this protected relational space, even within a broader institutional culture organised around visibility, surveillance, and continuous clinical accessibility.

Finally, the third theme—**Removal of Dirt and Impurities: Restoration of Bodily Order**—brings into focus the symbolic dimension of cleanliness in the ICU. Mary Douglas's (1966) classic work *Purity and Danger* reminds us that notions of dirt are not only about hygiene but also about symbolic order: ‘dirt is matter out of place.’ In the ICU, bodily fluids, dirty bedlinen, and unwashed skin can also be interpreted as signs of disorder, vulnerability, and disruption of the expected orderliness of the clinical environment. The bed‐bath, through the removal of these signs, restores the patient to a state of visible order and bodily presentation that aligns with the aesthetic and professional norms of the unit. This does not mean that cleanliness itself necessarily humanises care, since clinical procedures and objects must also be maintained in clean and orderly ways. Rather, the findings suggest that the meaning of cleanliness in nursing care lies in how bodily care is enacted: through attentiveness, touch, protection of exposure, and sustained engagement with the patient's vulnerable body.

As Tronto (1993) argues, care involves attentiveness and responsibility toward the needs of others. In our study, nurses enacted this attentiveness not only through the completion of hygiene tasks, but through the manner in which care was performed: adjusting sheets carefully, managing exposure, washing gently, and maintaining the patient's bodily presentation despite the pressures of routine care. The cleaned and ordered bed‐space therefore functioned not merely as a technical outcome of hygiene care, but also as a visible expression of ongoing nursing presence and attentiveness.

This restoration also had an organisational dimension. For nurses, the orderly presentation of the patient reflected professional competence and attentiveness, while completion of morning care prepared the patient and bed‐space for the clinical activities that followed, including medical examination. In each case, cleanliness functioned both clinically and symbolically, reinforcing the legitimacy of care practices and the orderliness of the unit.

Douglas's framework also illuminates the connection between cleanliness and institutional order. In our ICU, the removal of dirt was closely tied to the maintenance of the unit's routines, professional expectations, and visual order. Nurses played a central role in restoring this order through everyday bodily and environmental care. Previous studies of nursing work have similarly discussed how bodily care, despite its importance to patient well‐being, may occupy a less prominent position within institutional prestige hierarchies (Mol et al. 2010; Twigg 2000).

Our findings also resonate with Jeannette Pols (2013) ethnographic work on washing practices, which distinguishes between dignity as privacy and autonomy (*humanitas*) and dignity as embodied appearance, cleanliness, and presentation (*dignitas*). In our study, the morning bed‐bath similarly involved not only hygiene maintenance but also the preservation of bodily presentation, comfort, and social appearance within the ICU environment. Practices such as smoothing sheets, washing the face, arranging the bed‐space, or protecting bodily exposure reflected forms of attentiveness that extended beyond technical completion of the task itself. Rather than representing extraordinary ethical acts, these practices formed part of the ordinary, routinised work of ICU nursing, through which nurses attempted to preserve dignity and bodily integrity within the constraints of intensive care.

The study by Philippe et al. (2024), drawing on Tronto's ethics of care, conceptualises nursing care as relational work enacted within complex organisational and technological environments. This resonates with our findings, where the morning bed‐bath emerged as one of the few sustained moments of bodily proximity and attentiveness within the highly regulated ICU routine. Rather than suggesting overt resistance to institutional power, our findings indicate that nurses continued to enact relational and dignity‐preserving forms of care within the constraints of routinised clinical practice. Everyday acts such as washing, repositioning, or smoothing a sheet may appear mundane within the wider ICU environment, yet they remain important components of maintaining comfort, bodily integrity, and attentiveness to the patient as a person.

Our findings also resonate with broader discussions regarding the gendered nature and relative invisibility of nursing labour (Xiarchi et al. 2024). Routine bodily care activities such as bathing, oral hygiene, repositioning, and maintaining patient comfort are essential components of intensive care nursing, yet their contribution may be less readily visible alongside the highly technological and biomedical activities characteristic of ICU care. The morning bed‐bath observed in this study illustrates how substantial nursing work occurs through bodily proximity to the patient, encompassing hygiene and comfort alongside continuous observation, clinical assessment, and relational care.

In conclusion, far from being a mere technical procedure, the morning bed‐bath observed in this ICU can be understood as a practice situated at the intersection of biomedical imperatives, cultural values, and the embodied relations between nurse and patient. Viewed through the theoretical perspectives employed in this paper, the findings suggest that distinctions between ‘technical’ and ‘basic’ care may be less clear‐cut than these categories imply. The bath incorporates technical skill in handling lines and tubes, monitoring the patient's clinical condition, and preventing skin breakdown, while simultaneously involving fundamental human care through attention to comfort, dignity, and bodily integrity. As a recurrent and routinised practice, the morning bed‐bath therefore provides one setting in which clinical, relational, and symbolic dimensions of nursing care become visible, even though the routine nature of this work may also render these dimensions less readily recognised.

### 4.1 Limitations

This study offers valuable insights into the cultural, social, and moral dimensions of morning bed‐baths within a critical care setting, highlighting both its strengths and its limitations. A major strength lies in the ethnographic approach, which enabled an in‐depth exploration of the embodied, relational, and symbolic aspects of bodily care in an ICU in Greece. Prolonged engagement in the field over 2 years enhanced the credibility of the findings, while the use of multiple data collection methods—including participant observation, interviews, and ethnographic fieldnotes—contributed to the richness and depth of the analysis (Polit and Beck 2017; Nowell et al. 2017). The prolonged immersion of the researcher in the field also allowed observations to be revisited, contextualised, and interpreted over time, including the recognition of organisational and cultural patterns that had not been immediately apparent during everyday clinical practice as an ICU nurse. The integration of critical medical anthropology and phenomenological perspectives offered an interpretive lens through which routinisation, bodily care, symbolic order, and institutional organisation could be explored within the ICU setting.

However, certain limitations should also be acknowledged. The research was conducted in a single ICU, which may limit the transferability of findings to other critical care contexts, particularly across different cultural or organisational settings (Sandelowski 2004). As an ethnographic account of a culturally and organisationally situated practice, the findings are not intended to be generalisable to ICU nursing more broadly; rather, they offer a contextualised interpretation that may have relevance to comparable critical care settings. In addition, as with all ethnographic research, the possibility of observer bias cannot be fully excluded. The first author's dual role as an ICU nurse and ethnographer may have shaped both observation and interpretation. To address this, ethnographic fieldnotes were developed systematically following established qualitative and ethnographic approaches, reflexive notes were maintained throughout fieldwork, and emerging interpretations were discussed regularly with academic supervisors, including anthropological supervisors who were not nurses and were unfamiliar with ICU culture. This process supported critical reflection on assumptions, helped challenge insider interpretations, and strengthened analytic rigour.

The theoretical framing also represents one possible interpretation of the ethnographic material. Foucault's concept of discipline is useful for illuminating the temporal organisation, routinisation, and institutional structures shaping everyday ICU practice; however, its emphasis on disciplinary power may give less analytical attention to nurses’ capacity to exercise professional agency within these structures. The present findings also demonstrate nurses exercising embodied clinical knowledge, judgement, advocacy, and negotiation within routinised practice. Foucault is therefore employed as one interpretive lens rather than as an exhaustive explanation of ICU nursing practice. Similarly, the use of Bourdieu and Douglas foregrounds particular embodied and symbolic dimensions of practice, while other theoretical perspectives might produce different interpretations of the same clinical landscape.

A further limitation relates to the potential Hawthorne effect, since nurses were aware that participant observation was taking place. Nevertheless, the prolonged duration of fieldwork, the researcher's active participation in everyday care activities, the observation of multiple shifts and different staff members over time, and the routine nature of the observed practices likely reduced the extent to which behaviour could be consistently modified for observational purposes.

Finally, although formal participant observation concluded in 2014, anecdotal follow‐up until 2024 suggested that many aspects of the morning bed‐bath routine had remained relatively unchanged; however, these later observations were not part of the original systematic data collection and should therefore be interpreted cautiously.

## 5 Conclusion

This focused analysis of a broader ethnographic study suggests that the morning bed‐bath in the Greek ICU studied was far more than a routine hygiene task. It emerged as a complex and embodied nursing practice through which care, nursing expertise, professional identity, and organisational values were enacted within the temporal and spatial structures of this ICU. By analysing the three interrelated themes—Proximity and Synchronicity to the Patients Needs and Clinical Condition, Creation of a Zone of Safety and Privacy, and Removal of Dirt and Impurities—the study illuminated how the morning bed‐bath could function simultaneously as bodily care, organisational routine, and a site of nursing agency. Its repeated enactment did more than reproduce routine: through bodily proximity, sensory attentiveness, and coordinated action, nurses enacted clinical expertise, relational care, and professional agency.

The findings also suggest that routinisation may render some dimensions of bodily and relational nursing work less readily visible, while at the same time providing an organised context within which such care is accomplished. The study illustrated how nurses negotiated attentiveness, dignity, bodily exposure, and patient comfort within highly structured and time‐regulated ICU routines. These routines should not, however, be understood solely through the lens of discipline or professional hierarchy; the observations also revealed coordination of clinical work, embodied expertise, practical organisation, and nurses’ capacity to exercise judgement and agency within routine practice. At the same time, nurses’ everyday language, including references to ‘washing’ and ‘bed‐making the patients,’ suggested how the complexity of the practice could become obscured by its routine description.

As this was a contextually situated ethnography conducted in a single Greek ICU, these findings are not intended to be generalised to critical care nursing more broadly. They may, however, contribute to wider discussions concerning the visibility of nursing labour, the meanings attributed to bodily care, and the relationship between organisational routine and relational nursing practice. Rather than presenting the morning bed‐bath as an exceptional ethical act, this study suggests that its significance lies precisely in its ordinariness: within the setting studied, it constituted a routine yet highly skilled form of nursing work through which attentiveness, bodily care, clinical judgement, and patient dignity could be sustained within everyday ICU practice.

Rather than reinforcing a simple distinction between ‘technical’ and ‘basic’ nursing care, the findings suggest that these dimensions frequently intersect within the morning bed‐bath. Within the setting studied, the practice involved not only technical competence and clinical vigilance, but also forms of embodied knowledge, relational attentiveness, and organisational coordination. By drawing attention to these dimensions of an ordinary and recurrent ICU practice, this study offers one anthropological interpretation of the morning bed‐bath and contributes to discussions in nursing philosophy, critical care, and anthropology concerning bodily care, routine practice, professional agency, and relational work within technologically intensive healthcare environments.

## Author Contributions

**Stelios Parissopoulos:** conceptualization, methodology, data analysis, writing – original draft preparation, writing – review and editing. **Marianna Mantzorou:** writing – review and editing. **George Tsakonitis:** writing – review and editing. **Meropi Mpouzika:** writing – review and editing. **Eleni Papagaroufali:** methodology, supervision, validation, data analysis. All authors have read and agreed to the published version of the manuscript.

## Funding

The authors have nothing to report.

## Ethics Statement

Although the study involved healthcare professionals and did not involve patients or vulnerable populations, its design adhered to the ethical principles outlined in the Declaration of Helsinki, including voluntary participation, informed consent, and protection of participant confidentiality. The study was approved (a) by the PhD Program at the Department of Social Anthropology, PANTEION University of Social and Political Studies in Athens, and (b) by the Institutional Research and Ethics Committee of a hospital in Athens, where the study was conducted (protocol number 7‐11/7/12, date 11/7/12).

## Consent

Informed consent was obtained from all participant healthcare professionals involved in the study. An information leaflet was displayed in the unit informing staff and visitors that an observational study was in progress.

## Conflicts of Interest

The authors declare no conflicts of interest.

## Permission to Reproduce Material From Other Sources

Not applicable. All material presented in the article is original.

## Declaration of Generative AI and AI‐Assisted Technologies in the Writing Process

During the preparation of this work, the authors used **ChatGPT (OpenAI)** to assist with language refinement, structure, and clarity in drafting and revising the manuscript. After using this tool, the authors reviewed and edited the content as needed and take full responsibility for the content of the publication.

## Data Availability

Due to the sensitive and ethnographic nature of the field data, transcripts and fieldnotes are not publicly available. Extracts are included within the article where appropriate and anonymized in accordance with ethical requirements.
